# NLRC4 and NLRP3 in tissues as potential biomarkers for the diagnosis of non-small cell lung cancer in pulmonary tuberculosis

**DOI:** 10.3389/fcimb.2026.1872712

**Published:** 2026-08-26

**Authors:** Zetian Zhang, Zhikai Zhao, Shuanshuan Xie, Boyu Guo, Chenlei Cai, Chunyan Wu, Xiaohui Hao, Jian Ni, Haiyan Cui, Mengting Xiong

**Affiliations:** 1 Clinic and Research Center of Tuberculosis, Shanghai Pulmonary Hospital, School of Medicine, Tongji University, Shanghai, China; 2 Department of Pathology, Shanghai Pulmonary Hospital, School of Medicine,Tongji University, Shanghai, China; 3 Department of Respiratory Medicine, Shanghai Tenth People's Hospital, School of Medicine, Tongji University, Shanghai, China; 4 Department of Oncology, Shanghai Pulmonary Hospital, School of Medicine, Tongji University, Shanghai, China

**Keywords:** NLRC4, NLRP3, biomarkers, non-small lung cancer, pulmonary tuberculosis

## Abstract

**Background:**

Distinguishing non-small lung cancer (NSCLC) with tuberculosis from pulmonary tuberculosis (PTB) is still a major clinical problem. Misdiagnosis or missed diagnosis can lead to missed opportunities for optimal treatment, making highly sensitive diagnostic criteria crucial.

**Methods:**

Database analysis and histological analysis were used at the same time. A total of 55 patients were included in the histological analysis, including 20 patients with both NSCLC and PTB (NSCLC-PTB group), 20 patients with NSCLC alone (NSCLC group), and 15 patients with PTB alone (PTB group). The levels of NLRC4 and NLRP3 in each sample were analysed using the optical density method. Statistical analyses were performed, including the LIMMA algorithm, regression, and ROC analysis, to assess biomarker levels and their correlation with clinical outcomes.

**Results:**

The expression of NLRC4 and NLRP3 was significantly down-regulated in NSCLC tissues (both P < 0.001), and low expression of NLRC4 was associated with shorter overall survival (HR = 0.76, P < 0.001), while low expression of NLRP3 was associated with longer survival (HR = 1.21, P < 0.001). The level of NLRC4 in cancer nodules in the NSCLC-PTB group was significantly lower than that in the PTB group (0.0177 ± 0.0218 vs. 0.0299 ± 0.0117, *P* = 0.021). The level of NLRP3 in adjacent nodules in the NSCLC-PTB group was significantly higher than that in the PTB group (0.0398 ± 0.0282 vs. 0.0097 ± 0.0078, *P* < 0.001). At the same time, the results show that NLRC4 has the best diagnostic effect in squamous cell carcinoma, patients aged 60 and above, as well as NLRP3 in patients below 60, and the combination of NLRC4 and NLRP3 in adenocarcinoma.

**Conclusion:**

The use of NLRC4 and NLRP3 as biomarkers can improve the certainty of NSCLC prediction and has great prospects.

## 1 Introduction

Tuberculosis is a contagious respiratory disease caused by infection with Mycobacterium tuberculosis. Despite control efforts, it continues to have a high incidence and mortality rate, ranking among the top ten causes of death worldwide (Bagcchi, 2023; Yang et al., 2023). Tuberculosis has the potential to replace the coronavirus as the leading cause of death worldwide. According to the latest World Health Organization (WHO) guidelines, the low-complexity automated nucleic acid amplification test (NAATs) is the first choice for suspected cases, with positive bacteriological or molecular tests as the gold standard for diagnosis. Immunological tests such as purified protein derivative (PPD) or interferon-gamma release assay (IGRA) only indicate infection but cannot distinguish between active and inactive diseases (World Health Organization, 2025). According to the global cancer statistics of the WHO International Agency for Research on Cancer (ARC), lung cancer is now the leading cancer in terms of incidence and mortality in the world. The deaths caused by lung cancer in 2026 will exceed the combined total of colorectal cancer (ranked second) and pancreatic cancer (ranked third), with NSCLC accounting for more than 85% of all lung cancer cases (Wu et al., 2026). The literature shows that the incidence of tuberculosis and lung cancer is increasing year by year (Qin et al., 2022). There is also literature suggesting that patients with tuberculosis have a higher risk of lung cancer (Ho and Leung, 2018; Oh et al., 2020; Afifah et al., 2025; Zhou et al., 2025). However, due to numerous similarities in clinical manifestations and biomarkers between the two conditions, clinical diagnosis often presents challenges. For instance, both are systemic wasting diseases, typically presenting with symptoms such as cough, expectoration, chest pain, fever, fatigue, night sweats, and unexplained weight loss. In terms of imaging, both may show solitary pulmonary nodules, masses and cavities, ground-glass opacities, and enlargement of hilar and mediastinal lymph nodes. On positron emission tomography/computed tomography (PET/CT), both show high fluorodeoxyglucose (FDG) uptake (Bhatt et al., 2012; Keikha and Esfahani, 2018; Hwang et al., 2022). The current serological markers used to differentiate NSCLC from PTB are tumor markers, such as carcinoembryonic antigen (CEA), neuron-specific enolase (NSE), CA19-9, and CA125. However, they can also appear in other benign diseases, so their effect is limited when used alone. Currently, clinical practice combines CEA, cytokeratin 19 fragment (CYFRA21-1), and NSE detection to improve the detection rates (Preda et al., 2023). Therefore, new biomarkers with higher accuracy and speed are needed to assist in differentiation.

Small cell lung cancer (SCLC) is a high-grade neuroendocrine carcinoma that mainly occurs in current or former smokers, with an extremely poor prognosis. SCLC patients typically present with respiratory symptoms, including cough, shortness of breath (dyspnea), or hemoptysis. Imaging findings often appear at the center of the lungs, often accompanied by enlarged thoracic lymph nodes. Two-thirds of patients have distant metastatic disease at the time of initial diagnosis. Corresponding to the high metastatic tendency, the concentration of circulating tumor cells (CTCs) in SCLCs is the highest among all solid tumors. Genomic analysis of SCLC revealed extensive chromosomal rearrangement and high mutation burden, almost always accompanied by functional deactivation of the tumor suppressor genes tumor protein p53 (TP53) and retinoblastoma transcriptional corepressor 1 (RB1) (Rudin et al., 2021). Apart from a few very early-stage patients who may opt for a combination of surgery and adjuvant chemotherapy, the majority of SCLC treatments involve radiotherapy and chemotherapy. NSCLC can be further classified into lung adenocarcinoma, lung squamous cell carcinoma, and sarcomatoid tumors, with lung adenocarcinoma accounting for the largest proportion. An increasing number of studies show that NSCLC is not only associated with oncogenic gene mutations but also with other types of mutations, such as the absence or inactivation of tumor suppressor genes. As a transcription activator, SWI/SNF related, matrix-associated, actin-dependent regulator of chromatin, subfamily a, member 4 (SMARCA4) has diverse biological effects in the occurrence, metastasis, and resistance of NSCLC (Tian et al., 2023). Currently, the mainstream treatment for NSCLC is a comprehensive treatment based on surgery. But immune checkpoint inhibitors have been applied to non-small cell lung cancer in recent years (Tang et al., 2022). Although there have been many studies on early screening methods for lung cancer in recent years, advances in early detection have mainly focused on NSCLC, while effective early detection methods for SCLC remain lacking (Ely et al., 2026).

NLRC4 and NLRP3 inflammasomes, as important components of innate immunity, play a key role in a variety of disease processes (Zhang et al., 2025). The role of NLRC4 in antimicrobial defense has been fully established, but emerging evidence highlights its complex and often contradictory functions in cancer development and progression. NLRC4 exerts tumor suppression effects in colorectal and intestinal cancers and may be used for melanoma. NLRC4 also forms a unique tumor-inhibiting protein complex independently of NLR family apoptosis inhibitory proteins (NAIPs), apoptosis-associated speck-like protein containing a caspase recruitment domain (ASCs), and caspase-1, activating DNA damage responses. However, in obesity-related cancers and metastases, NLRC4 has tumor-promoting properties that promote pathological inflammation and angiogenesis (Zhang et al., 2026).At present, NLRC4 has been shown to have a positive correlation with the immune score of lung cancer and is associated with tumor suppression, making it a potential therapeutic target for lung cancer treatment (Xu et al., 2025). Single nucleotide polymorphisms (SNPs) found in genes encoding NLRC4 are associated with the risk of lung cancer (Jing et al., 2023). At the same time, functional SNPs in the NLRC4 gene can alter systemic and pulmonary inflammation, which is associated with lung inflammation and lung dysfunction in TB-infected individuals (Ravimohan et al., 2020). NLRP3 has been widely studied, regulating not only the tumor itself but also the composition of the tumor microenvironment (Deng et al., 2023). The NLRP3 level is elevated in multiple tumors, and some tumor cells can specifically activate NLRP3 in non-tumor cells. Overall, NLRP3 in the tumor microenvironment not only serves as a monitor of intracellular risk signals but may also be utilized by tumor cells to provide growth advantages or immune escape mechanisms by modulating inflammatory responses (Tengesdal et al., 2023). NLRP3 can promote the development of lung cancer by promoting the release of IL-1β and IL-18. Activation of NLRP3 inflammasomes contributes to the growth of Mycobacterium tuberculosis, thereby promoting the development of pulmonary tuberculosis (Feng et al., 2022; Palma Albornoz et al., 2023). Recent studies have shown extensive interactions among forms of programmed cell death (PCD), establishing the concept of PANoptosis. Oh S and others found that NLRP3, absent in melanoma 2 (AIM2), NLRC4, and Pylin form multi-protein complexes through interactions with ASCs, inducing PANoptosis in response to multiple inflammasome ligands. When NLRP3, AIM2, NLRC4, and Pylin are simultaneously activated by specific ligands, they form large multi-protein complexes with ASC, caspase-1, caspase-8, and receptor-interacting serine/threonine-protein kinase 3 (RIPK3), thereby inducing various forms of PCD. Additionally, these large multiprotein complexes are released into the extracellular space to function as multi-inflammatory bodies (Oh et al., 2023).

Currently, studies on NLRC4 and NLRP3 expression levels mostly use whole blood measurement. Whole blood assays offer several practical advantages, including minimal invasiveness, easy accessibility, and the requirement of only small volumes of blood. Additionally, unlike peripheral blood mononuclear cell (PBMC)-based assays that exclude neutrophils—the major leukocyte subset in peripheral blood—whole blood preserves caspase-1 activity in neutrophils and maintains native cellular interactions (Grinstein and Winkler, 2022). However, whole blood predominantly reflects the expression profile of circulating immune cells (monocytes, neutrophils, etc.) and cannot capture the expression of NLRC4 and NLRP3 in tissue-resident cells such as epithelial cells, fibroblasts, or parenchymal cells. Nor does it provide spatial information about differential expression across cell types or microenvironments. Besides, inflammasome components exhibit distinct tissue- and cell type-specific expression patterns. For instance, NLRC4 is expressed in the brain and macrophage-rich tissues, as well as in intestinal epithelial cells and phagocytes, while NLRP3 is present in various leukocytes and epithelial-type cells (Cahill and Humphries, 2025). Furthermore, inflammasome activation in the tissue microenvironment involves complex cellular interactions and local signaling that cannot be recapitulated in the circulation. Fewer studies have analyzed the differences between NLRC4 and NLRP3 in various nodules in PTB and NSCLC.

We hypothesized that the levels of NLRC4 and NLRP3 in tissues differed among different nodules in patients with PTB alone, NSCLC alone, and those with comorbidities. Additionally, we hypothesized that measurements of NLRC4 and NLRP3 in tissue can more accurately distinguish NSCLC from pulmonary tuberculosis than existing biomarkers, and that their combination is more effective. This study investigated the differences in the distribution of NLRC4 and NLRP3 in tuberculosis nodules, lung cancer nodules, and adjacent nodules in NSCLC combined with PTB, hoping to provide a more specific method for differentiating NSCLC from PTB and improving the accuracy and timeliness of diagnosis.

## 2 Methods

### 2.1 Clinical cohort

#### 2.1.1 Database study cohort

This study used public bioinformatics databases for exploratory analysis, including: GEPIA(Gene Expression Profiling Interactive Analysis; http://gepia2.cancer-pku.cn/), which is designed for cancer big data research and enables interactive analysis of gene expression levels; TIMER (https://cistrome.shinyapps.io/timer/) for systematic analysis of tumor-immune interactions, focusing on the association between tumor gene expression and patient survival prognosis; Kaplan–Meier Plotter(http://kmplot.com/analysis/index.php? P = background), which is commonly used for survival analysis, and the data sources cover GEO, EGA and TCGA databases. UALCAN (http://ualcan.path.uab.edu/analysis.html), based on the TCGA dataset, can perform tumor gene expression profile and survival analysis.

#### 2.1.2 Histology study cohort

A total of 55 participants were recruited in this study, including 20 patients with both NSCLC and PTB (NSCLC-PTB group), 20 patients with NSCLC alone (NSCLC group), and 15 patients with PTB alone (PTB group). All participants were from Shanghai Pulmonary Hospital in the past three years. The inclusion criteria for the disease group were: (1) Patients who has a confirmed diagnosis of PTB according to the international guidelines (a. Histological evidence (granuloma pathological features or acid-fast bacilli positivity); b. Positive tuberculosis molecular test)); (2) Patients who has confirmed pathological diagnosis of lung cancer; Clinical staging using the 7th edition of the TNM staging system authorized by the American Joint Committee on Cancer; (3) Patients who has detailed medical records. (4) Patients who have other tumors, suspected tuberculosis, or nontuberculous mycobacteria (NTMs) are excluded from the study. Before starting any anti-tuberculosis or antitumor therapy, including chemotherapy, radiotherapy, or targeted therapy, the patient is punctured for pathological examination at different sites of disease. The study was approved by the Ethics Committee, and informed consent was obtained from all patients.

### 2.2 Analysis methods

#### 2.2.1 Database analysis

In this study, the expression data of the target gene were obtained through the Gene Expression Profile module of the GEPIA database, and the screening conditions included: (1) the genes were NLRC4 and NLRP3; (2) LIMMA was used as the difference analysis method; (3)| Log2FC | threshold was set to 1; (4) the q-value threshold was set to 0.01. The expression amount was log2 (TPM + 1) on a logarithmic scale, covering a total of 33 tumor types such as ACC and BLCA. Subsequently, the expression differences between NLRC4 and NLRP3 in various tumor tissues and their adjacent normal tissues were simultaneously verified in the Exploration module of the TIMER database. For NSCLC, GEPIA’s Expression on Box Plots module was used to compare the expression differences between LUAD and LUSC tumor tissues and normal tissues, and the screening conditions were: (1) the genes were NLRC4 and NLRP3; (2)| Log2FC | threshold was 1; (3) the q-value threshold was 0.01. And the expression amount was also log2 (TPM + 1) on a logarithmic scale, and two isotypes of LUAD and LUSC were included for analysis.

The Kaplan–Meier Plotter database was used to evaluate the relationship between NLRC4 and NLRP3 expression levels and OS in NSCLC patients, and the specific screening conditions included: (1) the cancer type was limited to lung cancer; (2) the target genes were NLRC4 and NLRP3; (3) the survival outcome was OS. Finally, the above results were independently verified by the UALCAN platform (based on the TCGA database), and the screening conditions were: (1) cancer types included lung adenocarcinoma and lung squamous cell carcinoma; (2) the target genes were NLRC4 and NLRP3; (3) the patient grouping method was automatically selected with the best cut-off value.

Differential analysis of gene expression data was conducted using the LIMMA algorithm, with a corrected threshold set at q < 0.01. Samples were grouped by median gene expression levels, and survival curves were plotted using the Kaplan–Meier method. The hazard ratio (HR) was calculated based on the Cox proportional hazards model. In the survival analysis, the Log–rank test was used to assess the differences between groups, and a P value < 0.05 was considered statistically significant.

#### 2.2.2 Histology data analysis

**Figure 1:**
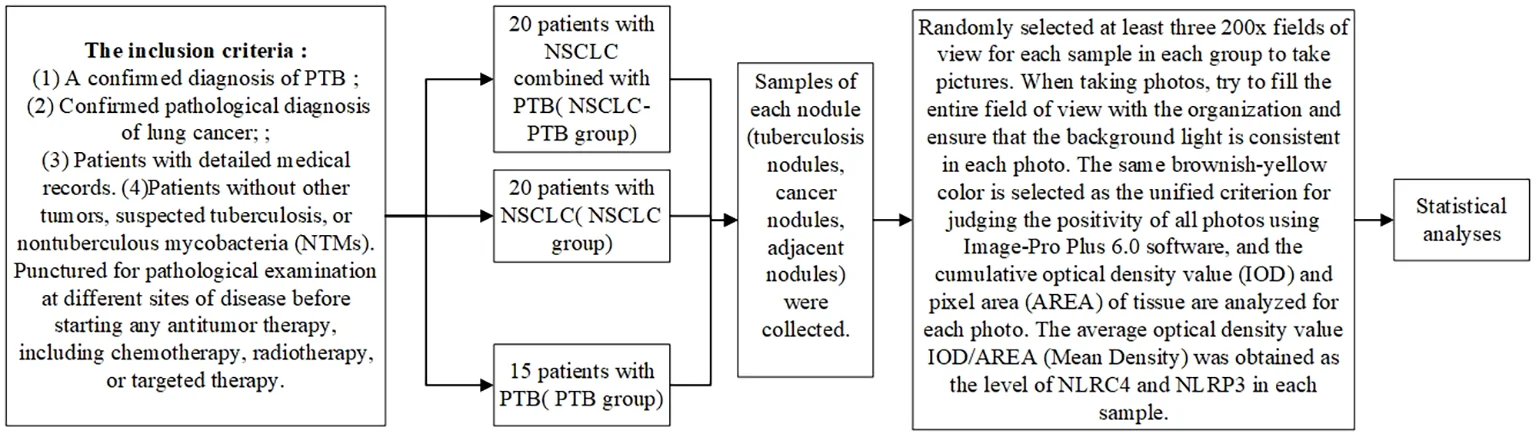
Technology roadmap.

Image-Pro Plus 6.0 (Media Cybernetics, Inc., Rockville, MD, USA) was used to obtain the mean density of immunohistochemistry of each sample. The specific analysis method is to randomly select at least three 200x fields of view for each slice in each group to take pictures. When taking photos, try to fill the entire field of view with the organization and ensure that the background light is consistent in each photo. The same brownish-yellow color is selected as the unified criterion for judging the positivity of all photos using Image-Pro Plus 6.0 software, and the cumulative optical density value (IOD) and pixel area (AREA) of tissue are analyzed for each photo. The average optical density value IOD/AREA (Mean Density) was obtained. NLRC4 and NLRP3 levels in different nodules of patients were determined based on pathological findings and analyzed using SPSS (version 20.0, IBM, Chicago, USA). Independent sample t-test, regression, ROC analysis, and multiple independent sample nonparametric tests were used to analyze the differences between groups. And the ROC curves were generated by SPSS and GraphPad Prism (version 10.6.0, GraphPad Software, USA). The technology roadmap is shown in **Figure 1**.

## 3 Results

### 3.1 Database-based NLRC4/NLRP3 expression characteristics and their relationship with patient prognosis

#### 3.1.1 Expression of NLRC4 and NLRP3 in NSCLC

**Figure 2:**
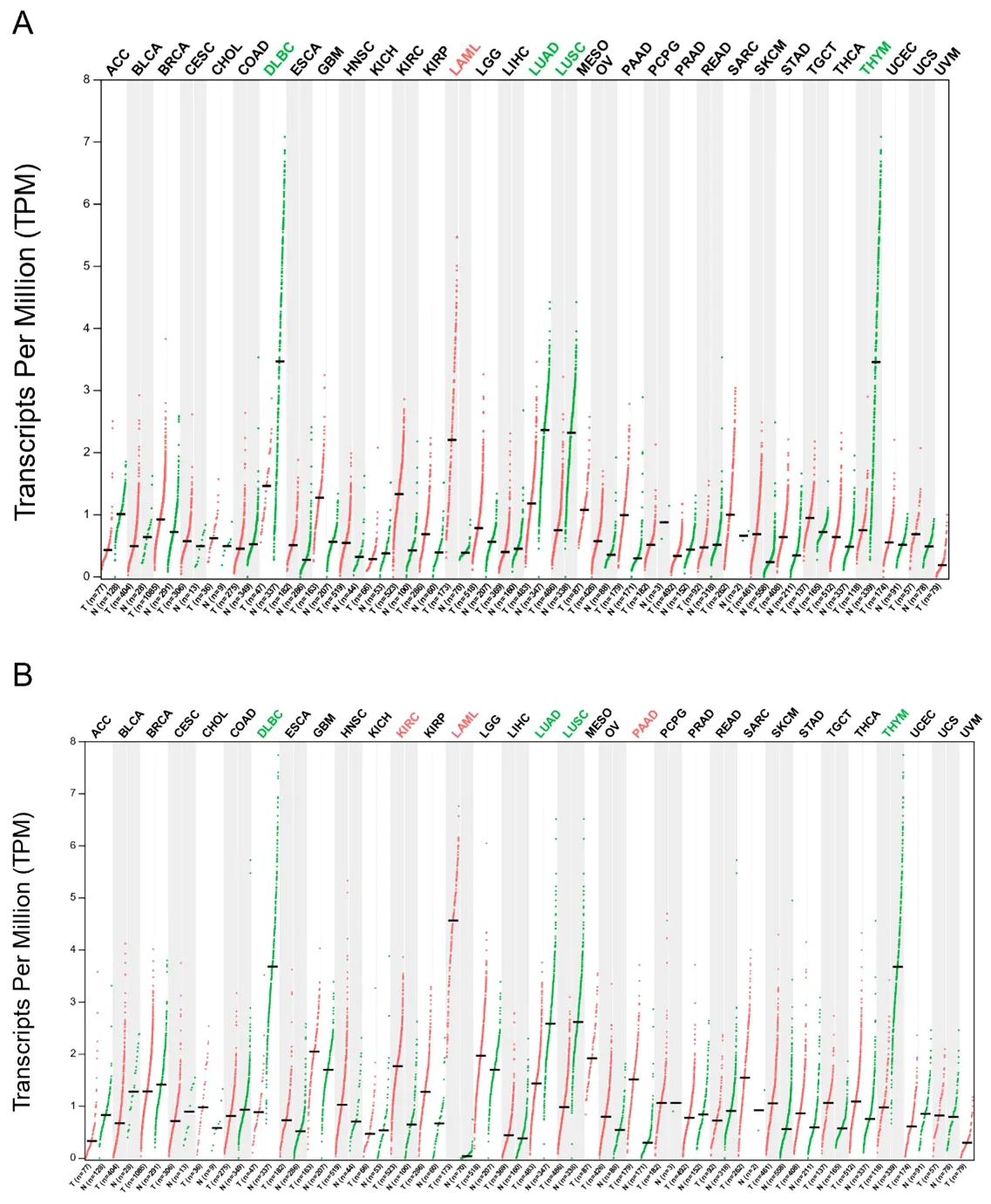
The expression differences of NLRC4 **(A)** and NLRP3 **(B)** in various malignant tumor tissues and their adjacent normal tissues in the GEPIA database.

**Figure 3:**
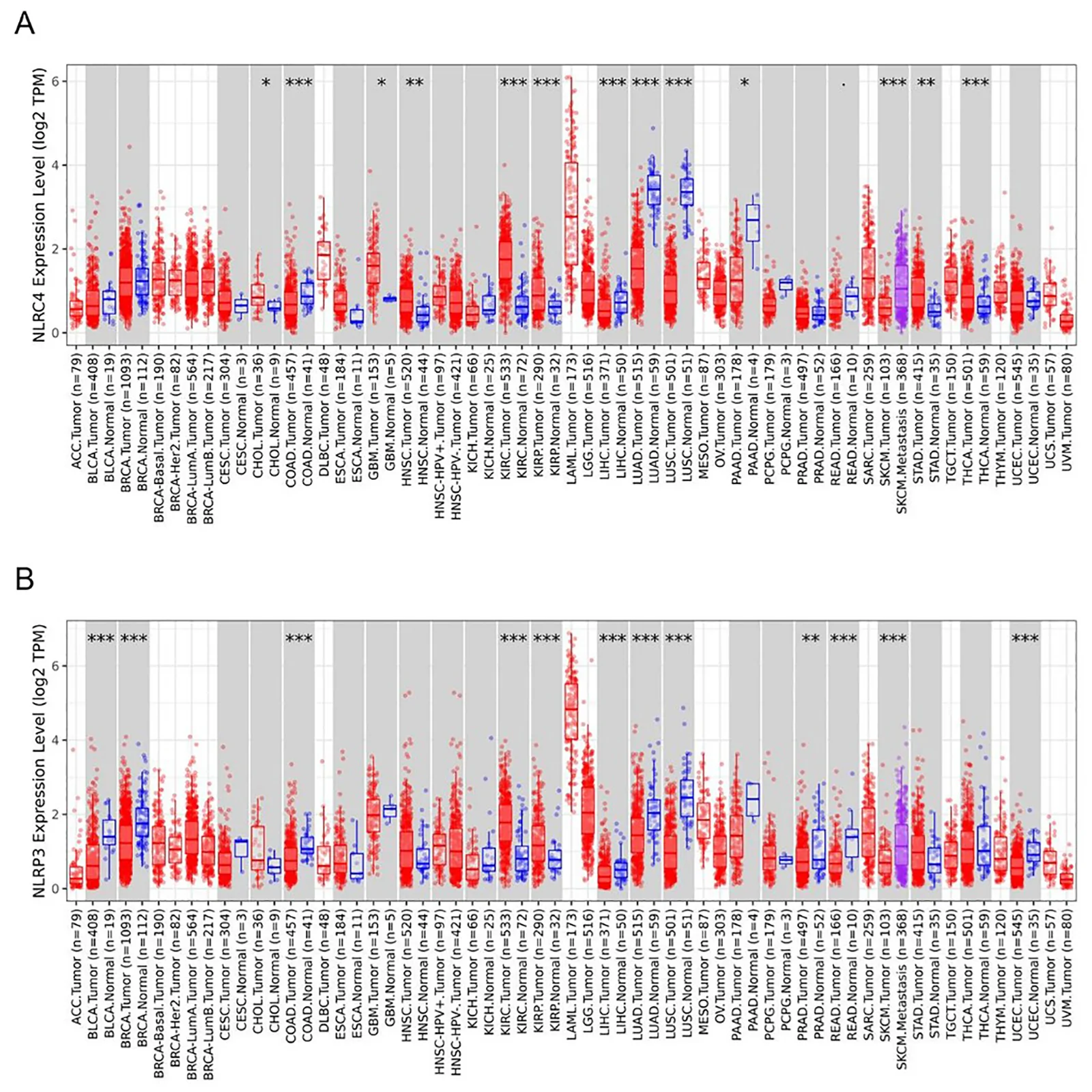
The expression differences of NLRC4 **(A)** and NLRP3 **(B)** in various malignant tumor tissues and their adjacent normal tissues in the TIMER database. ***means P < 0.001;**means P < 0.01;* means P < 0.05.

Analysis through the GEPIA database revealed that the expression level of the NLRC4 gene in acute myeloid leukemia cells was significantly higher than that in normal tissues (P < 0.05), while it was significantly lower in diffuse large B-cell lymphoma, lung squamous cell carcinoma, lung adenocarcinoma, and thymoma (P < 0.05). The expression analysis of the NLRP3 gene showed that it was higher in clear cell renal cell carcinoma, acute myeloid leukemia, and pancreatic cancer than in normal tissues (P < 0.05), but was downregulated in diffuse large B-cell lymphoma, lung squamous cell carcinoma, lung adenocarcinoma, and thymoma (P < 0.05) (**Figure 2**). The TIMER database further revealed that the NLRC4 gene was upregulated in cholangiocarcinoma, polyformative keratinocyte carcinoma, head and neck cancer, renal clear cell carcinoma, papillary renal cell carcinoma, gastric cancer, and thyroid cancer, but downregulated in colon cancer, liver cancer, lung adenocarcinoma, lung squamous cell carcinoma, and pancreatic cancer (**Figure 3A**). The NLRP3 gene was upregulated in clear cell renal cell carcinoma and papillary renal cell carcinoma, but downregulated in bladder urothelial carcinoma, invasive breast cancer, colon cancer, liver cancer, lung adenocarcinoma, lung squamous cell carcinoma, prostate cancer, rectal cancer, and endometrioid carcinoma (**Figure 3B**). Both database analyses indicated that the expression levels of NLRC4 and NLRP3 in lung adenocarcinoma and lung squamous cell carcinoma tissues were lower than those in adjacent normal tissues. Further verification of the expression differences of the target genes in lung adenocarcinoma and lung squamous cell carcinoma tumor tissues and adjacent normal tissues was conducted in the GEPIA database (**Figure 4.3**), and the results were consistent with the previous findings.

**Figure 4:**
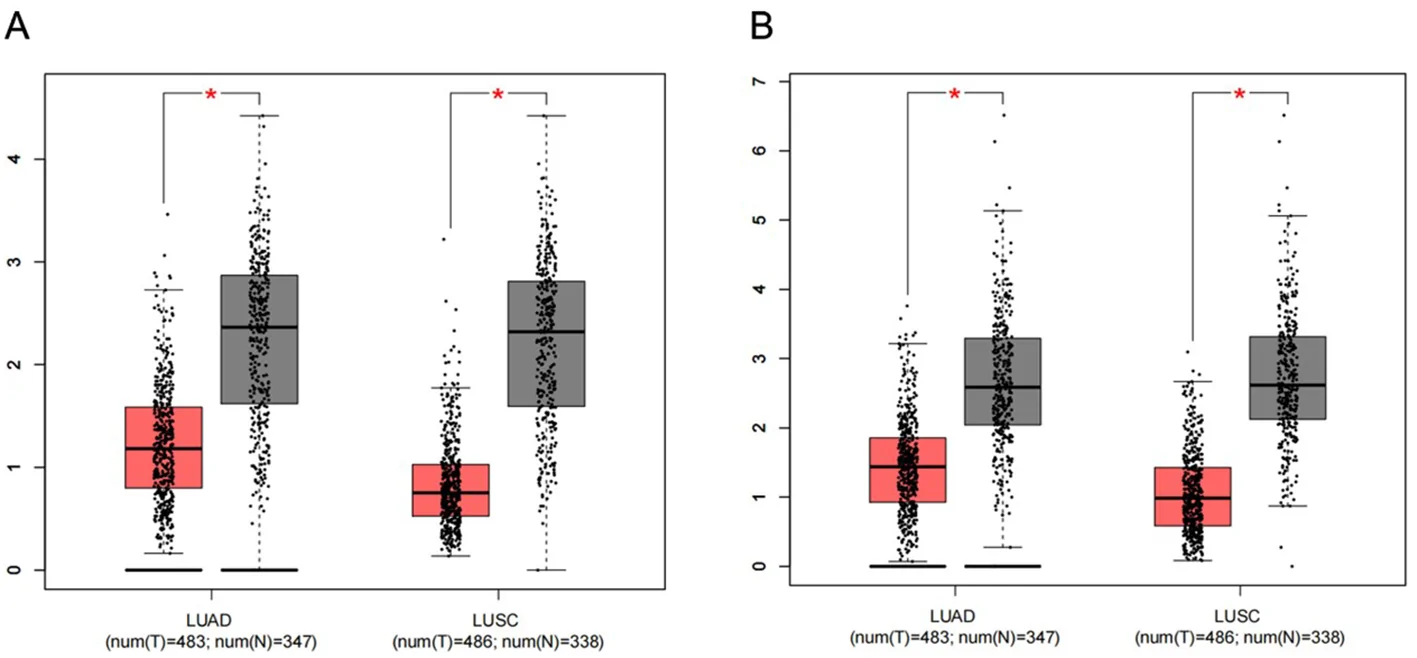
The expression differences of NLRC4 **(A)** and NLRP3 **(B)** in lung adenocarcinoma and lung squamous cell carcinoma tissues and their adjacent normal tissues in the GEPIA database. ***means P < 0.001; **means P < 0.01; * means P < 0.05.

**Figure 5:**
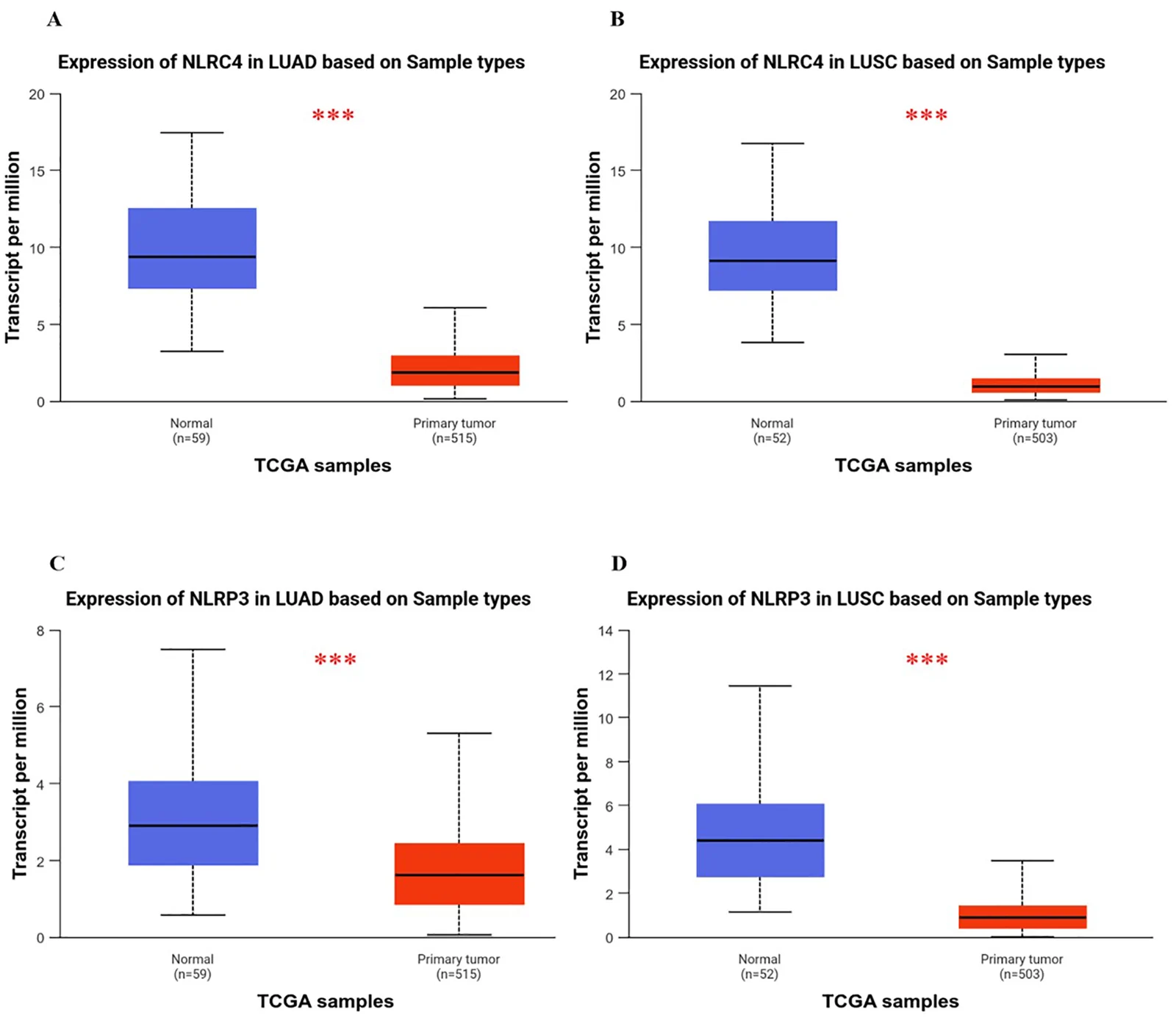
The expression differences of NLRC4 **(A)** LUAD; **(B)** LUSC and NLRP3 **(C)** LUAD; **(D)** LUSC in lung adenocarcinoma and lung squamous cell carcinoma tissues and their adjacent normal tissues in the UALCAN database. Red box packing diagram: tumor tissue; Blue box picture: normal organization. ***means P < 0.001; **means P < 0.01; * means P < 0.05.

The validation results from the UALCAN database also showed that in LUAD and LUSC, both NLRC4 (**Figures 5A, B**) and NLRP3 (**Figures 5C, D**) were expressed at low levels (P < 0.001), which was consistent with the analysis results from the GEPIA and TIMER databases.

#### 3.1.2 The effect of NLRC4 and NLRP3 gene expression levels on the prognosis of NSCLC patients

**Figure 6:**
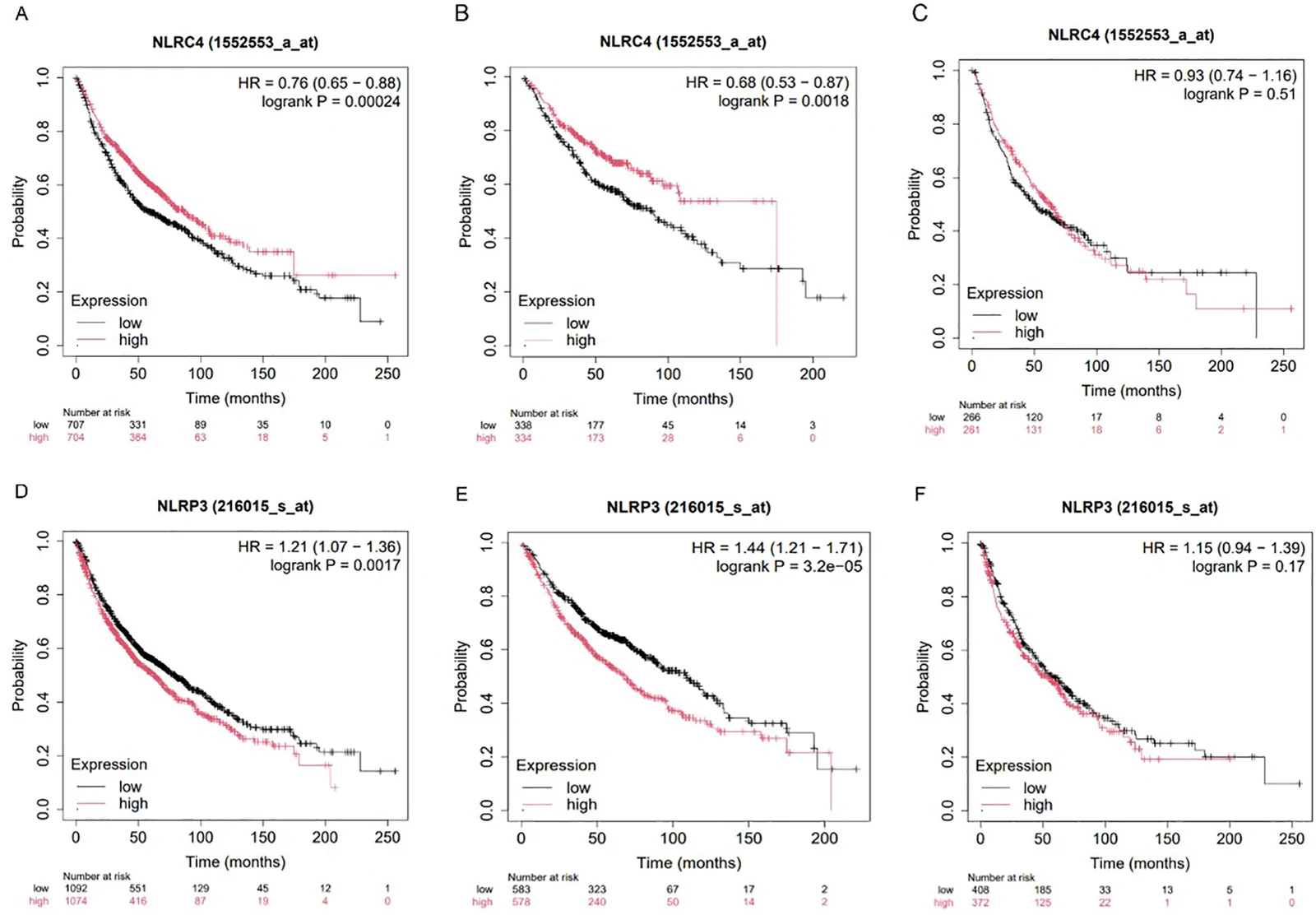
The Kaplan–Meier Plotter database illustrates the effects of NLRC4 and NLRP3 expression on the prognosis of patients with non-small cell lung cancer (NSCLC). High NLRC4 expression serves as a protective factor for NSCLC **(A)** and lung adenocarcinoma (LUAD) **(B)**; high NLRP3 expression acts as a risk factor for NSCLC **(D)** and LUAD **(E)**. Neither gene shows a statistically significant impact on the prognosis of lung squamous cell carcinoma (LUSC) **(C, F)**.

Kaplan–Meier Plotter database analysis showed that low expression of the NLRC4 gene was associated with shorter OS in NSCLC patients (HR = 0.76, 95% CI: 0.65–0.88, P < 0.001, **Figure 6A**), and the same trend was observed in the LUAD subtype (HR = 0.68, 95% CI: 0.53–0.87, P < 0.001, **Figure 6B**). In patients with LUSC, NLRC4 expression levels were not significantly different from OS (HR = 0.93, 95% CI: 0.74–1.16, P = 0.51, **Figure 6C**). NLRP3 gene analysis showed that the OS was higher in the low-to-moderate NSCLC group (HR = 1.21, 95% CI: 1.07–1.36, P < 0.001, **Figure 6D**), and that the survival of the low-expression group was also prolonged in the LUAD subtype (HR = 1.44, 95% CI: 1.21–1.71, P < 0.001, **Figure 6E**). There was no statistically significant difference in NLRP3 expression levels and OS in LUSC patients (HR = 1.15, 95% CI: 0.94–1.39, P = 0.17, **Figure 6F**).

**Figure 7:**
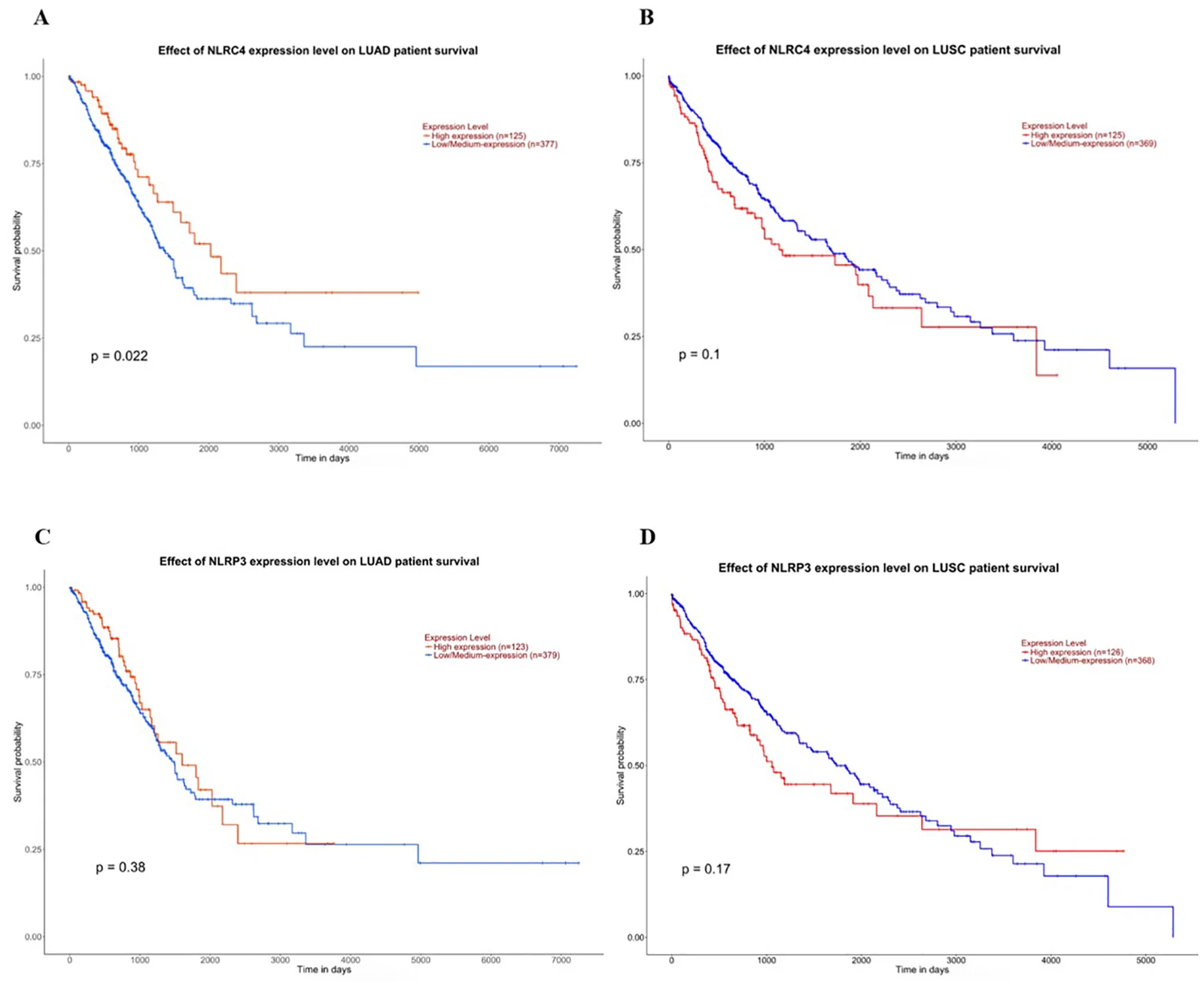
The influence of NLRC4 and NLRP3 expression on the prognosis of lung cancer patients was analyzed based on the UALCAN database. It shows that low NLRC4 expression was a risk factor for LUAD **(A)**, but had no significant association with prognosis in LUSC **(B)**; NLRP3 expression was not significantly associated with prognosis in either LUAD **(C)** or LUSC **(D)**.

Further survival analysis was performed using the UALCAN database, and the results showed that the overall survival of the low NLRC4 expression group in LUAD patients was significantly shortened (P = 0.022, **Figure 7A**), suggesting that low expression of NLRC4 may indicate poor prognosis. In LUSC patients, NLRC4 expression was not significantly associated with survival (P = 0.1, **Figure 7B**), which was consistent with the results of the Kaplan–Meier Plotter database. The expression level of the NLRP3 gene in LUAD (P = 0.38, **Figure 7C**) and LUSC (P = 0.17, **Figure 7D**) showed no significant correlation with overall survival, suggesting that the NLRP3 gene was not a prognostic factor for NSCLC. This part of the results differed from the results of the Kaplan–Meier Plotter database (**Figure 7E**).

### 3.2 The expression distribution of NLRC4/NLRP3 in lung tissue and its histological specificity

#### 3.2.1 Participants’ characteristics

Among the 20 patients with NSCLC combined with PTB, 9 were males, with a mean age of 58.93 ± 7.09 years. While among the 20 patients with NSCLC, 14 were male, with a mean age of 61.95 ± 10.16 years. Of the 15 patients with PTB alone, 11 were male, with a mean age of 59.73 ± 11.39 years. There was no significant difference in mean age between the groups (*P* = 0.612).

#### 3.2.2 Pathological NLRC4 levels of nodules in different groups

There was no significant difference between the NLRC4 levels of tuberculosis nodules in the NSCLC-PTB group (0.0273 ± 0.0218, *P* = 0.0049) and the levels of NLRC4 in tuberculosis nodules in the PTB group (0.0299 ± 0.0117, *P* = 0.0030), a power of 0.051 (1-β error probability). No significant difference between the NLRC4 levels of tuberculosis nodules in the NSCLC group (0.0168 ± 0.0154, *P* = 0.0027) and the NLRC4 levels of tuberculosis nodules in the NSCLC-PTB group (*P* = 0.05). The NLRC4 levels of tuberculosis nodules in the NSCLC group were significantly lower than those in the PTB group (*P* = 0.002). NLRC4 levels in tuberculous nodules of the NSCLC-PTB group showed no significant difference compared to those in cancer nodules (0.0177 ± 0.0218) or adjacent nodules (0.0247 ± 0.0148), *P* = 0.094, *P* = 0.674. And the NLRC4 levels of the cancer nodules in the NSCLC-PTB group were significantly lower than those in the PTB group (*P* = 0.021), but no significant difference between the adjacent nodules in the NSCLC group and the PTB group (*P* = 0.271). At the same time, there was no significant difference in NLRC4 levels between age < 60 years (0.0275 ± 0.0170) and age ≧ 60 (0.0207 ± 0.0169), with a power of 0.179.

#### 3.2.3 Pathological NLRP3 levels of nodules in different groups

There was no significant difference between the levels of NLRP3 in the NSCLC-PTB group (0.0247 ± 0.0148 and the levels of NLRP3 in the PTB group (0.0097 ± 0.0078), *P* = 0.243. The NLRP3 levels in the NSCLC group (0.0064 ± 0.0067) were significantly lower than the NLRP3 levels of tuberculosis nodules in the NSCLC-PTB group (*P* < 0.001). There was no significant difference between the NLRP3 levels in the NSCLC group and the NLRP3 levels in the PTB group (*P* = 0.189). NLRP3 levels in tuberculous nodules of the NSCLC-PTB group showed no significant difference compared to those in cancer tissue (0.0994 ± 0.0124) or pericancerous nodules (0.0398 ± 0.0282), *P* = 0.215, *P* = 0.262. There was no significant difference between the NLRP3 levels of cancer nodules in the NSCLC-PTB group and the PTB group (*P* = 0.945), but there was a significant difference between the adjacent nodules and the PTB group (*P* < 0.001). Additionally, NLRP3 levels showed no significant difference between patients aged < 60 years (0.0081 ± 0.0076) and those aged ≧ 60 (0.0114 ± 0.01019), *P* = 0.210.

#### 3.2.4 The correlation of NLRC4 levels with clinical parameters

We had examined the correlation between NLRC4 levels and clinical parameters. Univariate regression analysis showed that there was no significant correlation between NLRC4 levels of tuberculosis nodules and age and blood type in the NSCLC-PTB group (*P* = 0.695, *P* = 0.576). Significant correlation was observed between NLRC4 levels in NSCLC-PTB group tuberculous nodules and serum IGRA (*P* = 0.001). No significant correlation was found between NLRC4 levels in NSCLC-PTB group cancerous tissue and age or serum IGRA (*P* = 0.615, *P* = 0.541). NLRC4 levels in adjacent tissue of the NSCLC-PTB group showed no significant correlation with age or serum IGRA (*P* = 0.490, *P* = 0.507). NLRC4 levels in the PTB group showed no significant correlation with age (*P* = 0.639). And there was no significant correlation between NLRC4 level and age in the NSCLC group (*P* = 0.084).

#### 3.2.5 The correlation of NLRP3 levels with clinical parameters

We had also examined the correlation between NLRP3 levels and clinical parameters. Univariate regression analysis showed that there was no correlation between NLRP3 levels of tuberculosis nodules and age or serum IGRA in the NSCLC-PTB group (*P* = 0.526, *P* = 0.910). Similarly, there was no significant correlation between NLRP3 levels of cancer nodules and age or serum IGRA in the NSCLC-PTB group (*P* = 0.464, *P* = 0.963). NLRC3 levels in peritumoral tissue of the NSCLC-PTB group showed no significant correlation with age or serum IGRA (*P* = 0.842, *P* = 0.684). There was no significant correlation between NLRP3 levels and age in the PTB group (*P* = 0.970). And there was no significant correlation between NLRP3 levels and age in the NSCLC group (*P* = 0.130).

#### 3.2.6 The diagnostic value of NLRC4 and NLRP3 in different groups

**Figure 8:**
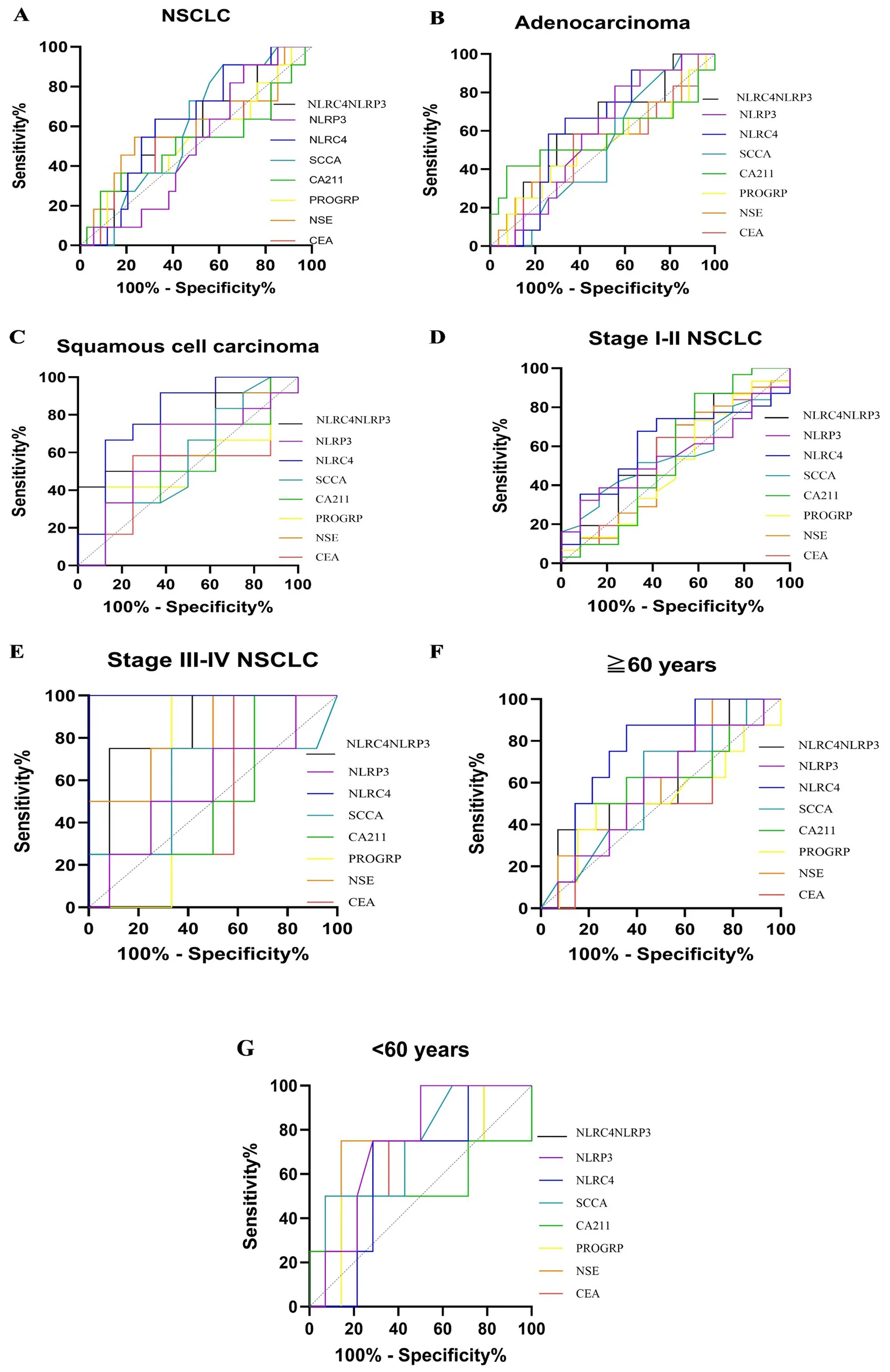
ROC curve of the diagnostic value of CEA, NSE, PROGRP, CA211, SCCA, NLRC4, NLRP3 and the combination of NLRC4 and NLRP3 in different groups. **(A)** ROC curve in the NSCLC. **(B)** ROC in the adenocarcinoma. **(C)** ROC curve in the squamous cell carcinoma. **(D)** ROC curve in the Stage I-II NSCLC. **(E)** ROC curve in the Stage III-IV NSCLC. **(F)** ROC curve in the ≥ 60 years. **(G)** ROC curve in the < 60 years.

**Figure 8** shows the ROC curve analysis results of NLRC4, NLRP3, the combination of NLRC4 and NLRP3, CEA, NSE, PROGRP, CA211, and SCAA for NSCLC diagnosis. NLRC4 had the highest diagnostic value for NSCLC (AUC: 0.626, 95% CI, 0.326-0.679; *P* = 0.326). Specifically, the combination of NLRC4 and NLRP3 had the best diagnostic effect in the prediction of adenocarcinoma (AUC: 0.615, 95%CI: 0.425-0.806). NLRC4 alone demonstrated the highest diagnostic accuracy for squamous cell carcinoma prediction (AUC: 0.802, 95% CI: 0.588–1.000; p = 0.025). For predicting stage I-II lung cancer, NLRC4 demonstrated the highest diagnostic value (AUC: 0.608; 95% CI: 0.428–0.789; p = 0.004), and it also showed the highest diagnostic value (AUC: 1.000; p = 0.278) for predicting stage III-IV lung cancer. NLRC4 had the highest diagnostic value in predicting lung cancer in patients aged ≧ 60 years (AUC: 0.740; 95% CI, 0.525-0.956; *P* = 0.070), while NLRP3 alone demonstrated the highest diagnostic value in patients < 60 years old (AUC: 0.741; 95% CI, 0.503-0.980; *P* = 0.152). Data concerning AUC, Standard error, P value, and 95%CI are detailed in **Table 1**.

**Table 1:** ROC curve of the diagnostic value of CEA, NSE, PROGRP, CA211, SCCA, NLRC4, NLRP3, and the combination of NLRC4 and NLRP3 in different groups.

	AUC	Standard error	P value	Lower limit of 95%CI	Higher limit of 95%CI
NSCLC					
CEA	0.561	0.107	0.544	0.352	0.771
NSE	0.594	0.109	0.355	0.380	0.807
PROGRP	0.535	0.104	0.731	0.330	0.739
CA211	0.511	0.113	0.916	0.289	0.733
SCCA	0.588	0.087	0.383	0.417	0.759
NLRC4	0.626	0.089	0.214	0.451	0.800
NLRP3	0.503	0.090	0.979	0.326	0.679
NLRC4+NLRP3	0.580	0.097	0.428	0.389	0.771
Adenocarcinoma					
CEA	0.516	0.105	0.875	0.311	0.721
NSE	0.580	0.108	0.432	0.369	0.791
PROGRP	0.487	0.107	0.900	0.227	0.698
CA211	0.436	0.118	0.530	0.204	0.667
SCCA	0.510	0.094	0.925	0.326	0.693
NLRC4	0.609	0.094	0.286	0.425	0.793
NLRP3	0.603	0.093	0.315	0.420	0.785
NLRC4+NLRP3	0.615	0.097	0.258	0.425	0.806
Squamous cell carcinoma					
CEA	0.510	0.140	0.939	0.235	0.786
NSE	0.573	0.136	0.589	0.306	0.840
PROGRP	0.448	0.133	0.700	0.187	0.709
CA211	0.521	0.137	0.877	0.253	0.789
SCCA	0.562	0.140	0.643	0.288	0.837
NLRC4	0.802	0.109	0.025	0.588	1.000
NLRP3	0.396	0.136	0.440	0.130	0.662
NLRC4+NLRP3	0.677	0.123	0.190	0.437	0.917
Stage I-II NSCLC					
CEA	0.525	0.102	0.802	0.325	0.725
NSE	0.547	0.107	0.636	0.338	0.757
PROGRP	0.497	0.105	0.978	0.292	0.703
CA211	0.444	0.114	0.578	0.221	0.668
SCCA	0.565	0.090	0.513	0.389	0.742
NLRC4	0.608	0.092	0.278	0.428	0.789
NLRP3	0.572	0.091	0.469	0.394	0.750
NLRC4+NLRP3	0.600	0.097	0.316	0.409	0.791
Stage III-IV NSCLC					
CEA	0.438	0.168	0.716	0.108	0.767
NSE	0.813	0.125	0.069	0.567	1.000
PROGRP	0.333	0.136	0.332	0.067	0.600
CA211	0.542	0.171	0.808	0.206	0.877
SCCA	0.406	0.191	0.585	0.032	0.780
NLRC4	1.000	0.000	0.004	1.000	1.000
NLRP3	0.417	0.170	0.628	0.084	0.749
NLRC4+NLRP3	0.854	0.107	0.039	0.645	1.000
≧ 60 years					
CEA	0.452	0.138	0.717	0.182	0.722
NSE	0.635	0.130	0.311	0.380	0.889
PROGRP	0.524	0.141	0.856	0.247	0.801
CA211	0.413	0.136	0.515	0.147	0.680
SCCA	0.428	0.129	0.587	0.176	0.680
NLRC4	0.740	0.110	0.070	0.525	0.956
NLRP3	0.462	0.133	0.772	0.201	0.722
NLRC4+NLRP3	0.635	0.127	0.311	0.386	0.883
< 60 years					
CEA	0.714	0.157	0.203	0.406	1.000
NSE	0.732	0.166	0.167	0.407	1.000
PROGRP	0.375	0.160	0.457	0.062	0.688
CA211	0.482	0.206	0.915	0.078	0.886
SCCA	0.714	0.140	0.203	0.440	0.989
NLRC4	0.625	0.144	0.457	0.342	0.908
NLRP3	0.741	0.122	0.152	0.503	0.980
NLRC4+NLRP3	0.679	0.161	0.288	0.363	0.995

## 4 Discussion

Studies have shown that tuberculosis is an independent risk factor for lung cancer, and the mortality rate of patients with both tuberculosis and lung cancer is 2.36 times that of patients with lung cancer alone (Moon et al., 2023). However, in the process of clinical diagnosis and treatment, lung cancer and tuberculosis usually have similar clinical symptoms and imaging characteristics, which can easily lead to delays in diagnosis and treatment, resulting in missed opportunities for diagnosis and treatment. Conventional tumor indicators are not accurate and sensitive enough in diagnosing lung cancer patients with tuberculosis. Therefore, there is an urgent need to discover new indicators to distinguish lung cancer from pulmonary tuberculosis.

The activation of NLRC4 inflammasomes mainly relies on pathogenic bacteria, which can be recruited by NAIP and assembled to form NAIP-NLRC4 inflammasomes. NAIP binds to specific ligands to promote NLRC4 oligomerization, recruiting ASCs and caspase-1to form the NAIP-NLRC4 inflammasome complex, which is subsequently activated to induce downstream inflammatory responses (Miao et al., 2010; Wang et al., 2021; Fu et al., 2024). This process is essential for mounting an immune response against tuberculosis infection. In addition, studies have confirmed the role of NLRC4 in lung adenocarcinoma. Low expression of NLRC4 leads to reduced production of cytokines and chemokines, such as C-X-C motif chemokine ligand 9 (CXCL9), CXCL10, CXCL16, and CCL5, resulting in decreased immune cell infiltration. While tissues with high NLRC4 expression have more infiltrating immune cells, such as CD4^+^ T cells, CD8^+^ T cells, Treg cells, DC cells, macrophages, NK cells, and CD20^+^ B cells. These immune cells play an important role in the regulation of cancer (Vilariño et al., 2020; Jing et al., 2023). Its activation is closely related to the occurrence of a variety of diseases, especially in chronic inflammation and autoimmune diseases. NLRP3 activation usually triggers the release of inflammatory mediators by sensing danger signals within cells, such as cell damage and pathogen infection, promoting the occurrence of inflammatory responses (Zhivaki and Kagan, 2021; Tengesdal et al., 2023). Activated NLRP3 inflammasomes further enhance local and systemic inflammatory responses by promoting the maturation and secretion of IL-1β and IL-18, which also promote the proliferation and metastasis of tumor cells. In addition, NLRP3 inflammasomes also play a key role in the immune escape of lung cancer cells. Tumor cells activate NLRP3 inflammasomes to promote IL-1β release and drive macrophage polarization toward the M2 type, thereby forming an immunosuppressive microenvironment that evades immune surveillance by the host (Ben-Sasson et al., 2013; Gu et al., 2022; Zhu et al., 2024). The activation of NLRP3 inflammasomes is also related to the epithelial-mesenchymal transition process of tumor cells, further enhancing their invasiveness and metastasis potential (Chen et al., 2020; Liu et al., 2022).

This study systematically evaluated the expression characteristics of NLRC4 and NLRP3 inflammasomes in NSCLC patients with PTB and their application value as potential diagnostic biomarkers. Database analysis showed that the expression of NLRC4 and NLRP3 was significantly down-regulated in NSCLC tissues (both P < 0.001), and low expression of NLRC4 was associated with shorter overall survival (HR = 0.76, P < 0.001), while low expression of NLRP3 was associated with longer survival (HR = 1.21, P < 0.001). Tissue validation demonstrated that there were significant differences in NLRC4 and NLRP3 expression across groups and between different nodules, which suggested the potential of NLRC4 and NLRP3 as biomarkers for tuberculosis-associated lung cancer.

In this study, it was found that low expression of NLRC4 leads to shorter overall survival, while low expression of NLRP3 is associated with longer survival. This suggests that NLRC4 may exert a tumor suppressive effect inside lung cancer cells, while NLRP3 may have a pro-tumor effect in lung cancer. The expression level of the NLRP3 gene in LUAD (P = 0.38, **Figure 7C**) and LUSC (P = 0.17, **Figure 7D**) showed no significant correlation with overall survival, suggesting that the NLRP3 gene was not a prognostic factor for NSCLC. This part of the results differed from the results of the Kaplan–Meier Plotter database. There have been serological studies demonstrating that Mycobacterium tuberculosis infection significantly alters systemic NLRP3 levels, which is consistent with the conclusions of this study (Xiong et al., 2025). Whether tuberculosis infection is combined may be the reason for the differing conclusions of the two databases. It was found at the organizational level that the levels of NLRC4 in cancer nodules in patients with NSCLC combined with PTB were significantly lower than those in patients with PTB alone, while the levels of NLRP3 in adjacent nodules were significantly higher than those in patients with PTB alone. At the same time, the levels of NLRC4 and NLRP3 in NSCLC patients were significantly lower than those in PTB patients, while NLRC4 and NLRP3 in patients with NSCLC combined with PTB were at intermediate levels. This phenomenon arises because chronic infection caused by Mycobacterium tuberculosis elevates the level of inflammasomes, whereas tumor-mediated inflammasome suppression reduces NLRC4 and NLRP3 expression. The results also show that NLRC4 has the best diagnostic effect in squamous cell carcinoma, patients aged 60 and older, as well as NLRP3 in patients below 60, and the combination of NLRC4 and NLRP3 in adenocarcinoma. Both are better than traditional lung cancer diagnosis biomarkers such as CEA.

Our research has the following advantages: Firstly, we performed database analysis and histological analysis at the same time, which resulted in a larger sample size and greater clinical applicability. Secondly, we measured the levels of NLRC4 and NLRP3 in different groups of nodules by optical density method. And at the same time, three randomly selected regions were averaged for each nodule, so that the data were more representative. Finally, our research subjects are more targeted, selecting patients with NSCLC combined with PTB as the experimental group, while those with either lung cancer or tuberculosis alone served as controls, thereby sharpening the research focus.

Of course, there are some limitations to our research. The first is that the tissue sample size is relatively small and comes from a single study center, which may reduce the universality of the sample. Furthermore, we did not consider the potential impact of other factors on the experiment. Finally, this study lacks organizational molecular quantification, which is an area that needs to be covered in future studies.

## 5 Conclusion

NLRC4 and NLRP3 in tissues are promising biomarkers for distinguishing NSCLC complicated by pulmonary tuberculosis from those with pulmonary tuberculosis alone, and have higher diagnostic accuracy than traditional lung cancer markers.

## Data Availability

The original contributions presented in the study are includedin the article/supplementary material. Further inquiries can bedirected to the corresponding author.
